# Exploring the Potential Driver Gene Mutations That Promote Renal Cancer Cell Metastasis and Implantation Based on Circulating Tumor Cells Culture

**DOI:** 10.3390/diagnostics13111855

**Published:** 2023-05-25

**Authors:** Baoan Hong, Xuezhou Zhang, Xin Du, Dazhi Yang, Zhiyuan Hu, Xiuli Zhang, Ning Zhang

**Affiliations:** 1 Key Laboratory of Carcinogenesis and Translational Research (Ministry of Education/Beijing), Department of Urology, Peking University Cancer Hospital & Institute, Beijing 100142, China; hbaurology@bjmu.edu.cn (B.H.); dxdoctor@126.com (X.D.); 2 Department of Urology, Beijing Anzhen Hospital, Capital Medical University, Beijing 100029, China; zhangxuezhou1128@163.com; 3 Acrogenic Biotechnologies INC, Rockville, MD 20850, USA; dazhiyang1211@gmail.com; 4 CAS Key Laboratory for Biomedical Effects of Nanomaterials and Nanosafety, CAS Key Laboratory of Standardization and Measurement for Nanotechnology, CAS Center for Excellence in Nanoscience, National Center for Nanoscience and Technology of China, Beijing 100190, China; huzy@nanoctr.cn

**Keywords:** renal cell carcinoma, circulating tumor cell, culture in vitro, CDX model, prognosis

## Abstract

Studies have shown that the circulating tumor cell (CTC) is a necessary condition for the invasion and distant metastasis of renal cell carcimona (RCC). However, few CTCs-related gene mutations have been developed which could promote the metastasis and implantation of RCC. The objective of this study is to explore the potential driver gene mutations that promote RCC metastasis and implantation based on CTCs culture. Fifteen patients with primary mRCC and three healthy subjects were included, and peripheral blood was obtained. After the preparation of synthetic biological scaffolds, peripheral blood CTCs were cultured. Successful cultured CTCs were applied to construct CTCs-derived xenograft (CDX) models, followed by DNA extraction, whole exome sequencing (WES) and bioinformatics analysis. Synthetic biological scaffolds were constructed based on previously applied techniques, and peripheral blood CTCs culture was successfully performed. We then constructed CDX models and performed WES, and explored the potential driver gene mutations that may promote RCC metastasis and implantation. Bioinformatics analysis showed that KAZN and POU6F2 may be closely related to the prognosis of RCC. We successfully performed the culture of peripheral blood CTCs and, on this basis we initially explored the potential driver mutations for the metastasis and implantation of RCC.

## 1. Introduction

Renal cell carcinoma (RCC) is one of the most common malignant tumors in the urinary system. The incidence rate of RCC accounts for about 4% of adult malignancy, and it is the sixth most common malignant tumor in men and the ninth in women in 2023 worldwide statistics [1]. Approximately 25% RCC patients already have local or distant metastatic lesions when diagnosed due to the lack of noticeable clinical symptoms at the early stage, and more than 20% of RCC patients after surgery still have recurrence and metastasis [2,3,4]. RCC is not sensitive to chemotherapy, and with the in-depth exploration of the molecular mechanism of RCC, the treatment of metastatic renal cell carcinoma (mRCC) has undergone cytokine therapy, molecular targeted therapy and immunotherapy [5]. In recent years, targeted therapy and immunotherapy have brought a new dawn to mRCC patients, while they still face the difficulties of drug resistance and treatment failure [6,7]. The prognosis for mRCC patients is unsatisfactory with a five-year survival rate remaining less than 20% while metastasis is the main cause of death for RCC patients [8]. Therefore, it is urgent and challenging to further explore the molecular mechanism of metastasis and implantation in RCC for diagnosis and treatment.

Recent studies have shown that the circulating tumor cell (CTC) is a necessary condition for the invasion and distant metastasis of RCC, which helps to clarify the mechanism of metastasis, guide treatment, and provide important references for judging its efficacy and prognosis [9,10,11]. CTCs refer to a kind of cell in the human body that is separated from the primary tumor focus and enters the peripheral blood circulation system; these cells circulate with the blood and can form new metastasis lesions after being separated from the blood circulation [12]. After entering the blood circulation, some CTCs can escape from the immune surveillance and finally enter the microenvironment of other organs. A small number of CTCs separated from the blood circulation can safely pass the quiescent period and eventually form new metastatic lesions under the action of various cytokines [13,14]. However, few CTCs-related gene mutations have been developed which could promote metastasis and the implantation of RCC patients.

Here in this study, the cationic polymer biomaterials developed by our team were used as biological scaffolds for 3D cell culture, and the CTCs of newly diagnosed patients with mRCC were successfully cultured and expanded in vitro for the first time in the world. The CTCs were inoculated subcutaneously into nude mice to construct the CTCs derived xenograft (CDX) model, and then whole exome sequencing (WES) was performed on the successfully cultured CTCs and the CDX models including their matched primary RCC tissues. The aim of this study is to identify the gene mutation on the molecular level during the transformation of primary RCC into the CTCs state and the formation of the CDX model by CTCs implantation through comparative analysis and cluster analysis, so as to explore the potential molecular drivers for promoting the metastasis and implantation of RCC.

## 2. Materials and Methods

### 2.1. Patients and Healthy Controls

The study was approved by the Medical Ethics Committee of Peking University Cancer Hospital (approval code: 2020KT21, approval date: 27 March 2020) and conducted following Good Clinical Practice and the Declaration of Helsinki. Informed consent was acquired from all patients. This study included 15 patients from Beijing Cancer Hospital and the inclusion criteria include: (1) patients with untreated primary mRCC; (2) clear cell renal cell carcinoma (ccRCC) confirmed by histology or cytopathology; (3) TxNxM1: evaluation according to the TNM staging standard of the American Joint Committee on Cancer (AJCC), 8th edition [15]; (4) ≥18 years old. Exclusion criteria include any one or more of the following: (1) patients received immunotherapy in the past four weeks; (2) patients received radiotherapy in the past two weeks, except for those who still have metastasis outside the radiation focus; (3) patients had other cancers or a history of cancer in the past five years. Detailed clinicopathological data of the 15 ccRCC patients are presented in Table 1. This study also recruited three healthy subjects as the control group and the inclusion criteria include: (1) the physical examination report within the past three months could be provided, and the report results show that no tumor and blood cell value is within the normal range; (2) ≥18 years old. Exclusion criteria include any one or more of the following: (1) subjects having hyperlipemia, hypertension, hyperglycemia, etc.; (2) subjects received chemotherapy or immunotherapy for any reason in the past four weeks; (3) subjects have a history of tumor or family genetic history.

### 2.2. Preparation of Synthetic Biological Scaffold

For the preparation of synthetic biological scaffolds, we used the methods previously reported [16,17]. In short, the selectively oxidized 2,3-dialdehyde cellulose is covalently crosslinked with polyamine polymer and the double bond of carbon-amino imine is reduced. Importantly, 2,3-dialdehyde cellulose is covalently crosslinked with functional block polymers to form polyamine-cellulose copolymers with three-dimensional dense interlocking networks. The amine group is protonated in an aqueous environment with pH < 9, so that the positively charged copolymer scaffold forms a hydrogel matrix. Fourier transform infrared spectrometer (FTIR spectrometer) was used to evaluate the functional group characterization of the material. The details of this synthetic biomaterial can be found in the US patent “Microcarriers, Matrices and Scaffolds for Culturing Mammalian Cells and Methods of Manufacture” (ID: US-20180094080-A1, April 2018) [18]. 

### 2.3. Culture of Peripheral Blood CTCs of RCC Patients

Peripheral blood from elbow veins was collected from 15 newly diagnosed mRCC patients. All patients were registered according to previously determined standards and signed the informed consent. Some 15 mL of peripheral blood was drawn from each patient and all samples were collected in a sterile EDTA-coated vacuum blood collection tube (BD Vacutainer), stored at 4 °C and sent to the laboratory for experimental treatment. The blood samples were processed within 60 min of collection.

The blood samples were mixed with 1×phosphate buffer saline (PBS) at room temperature in a 1:1 volume ratio, and then the samples were evenly divided into two 50 mL test tubes, each containing 15 mL Ficoll-Paque (Cytova Life Sciences, Marlborough, MA, USA cat no.17-1440-02); we took care not to mix the sample with Ficoll-Paque. The sample was rotated for 30 min at a speed of 800× *g* at room temperature with the centrifugal parameters adjusted for the minimum acceleration and no deceleration. After centrifugation, four layers were formed, including a plasma layer, a mononuclear cell layer, a Ficoll-Paque layer, and an erythrocyte layer. The plasma layer and mononuclear cell layer were collected and resuspended to a final volume of 50 mL using 1 × HBSS, and centrifuged at 4 °C for 10 min at a speed of 300× *g*. They were resuspended using 1 × HBSS and centrifuged again at 4 °C for 10 min at a speed of 300× *g*. The cells were resuspended in the stem cell culture medium which was mixed with the synthetic biological scaffold at a 1:1 volume ratio. Each treated sample was inoculated into multiple holes of 96-hole ultra-low absorption plate (approximately 15 mL blood was distributed into five or six holes) and cultured in a 37 °C, 5% CO_2_ moist incubator. The medium was changed every three days for a fresh medium. After one month of incubation, the cultures were treated for DNA extraction or grown for CDX.

### 2.4. Handling of Blood from Healthy Subjects

Two tubes of 7.5 mL each of blood were drawn from three healthy subjects, and the samples were treated as in the previous section for CTCs. Two of the three healthy subjects were male and one was female.

### 2.5. Application of Cultured CTCs to Construct CDX Models

Female six- to eight-week-old NPSG mice were purchased from the HFK Bioscience Co., Ltd. (Beijing, China) and maintained in accordance with the Beijing Experimental Animal Management Regulations. The laboratory was under specific pathogen-free (SPF) conditions. Mice were housed in a 12 h light/12 h dark setting, at 21 °C and 50% relative humidity, and were allowed free access to water and food.

Single cells derived from CTCs were resuspended in PBS/Matrigel mix (BD Biosciences, 1:1 volume) and injected into the subcutaneous tissue of the right back side of the mice near the axilla using a 27-gauge needle at a dose of 125,000 cells/site. The xenografts were collected within two months of the implantation. Four CTCs-derived xenograft (CDXs) models were eventually constructed by applying CTCs. The mice were anesthetized and executed, and fresh tumor tissue was collected according to the American Veterinary Medical Association guidelines.

### 2.6. DNA Extraction, Whole Exome Sequencing and Analysis

Cultures of CTCs from four patients and their matched tumor tissues were used for DNA extraction and Whole Exome Sequencing (WES). DNA was extracted from tumor tissues, peripheral blood leukocytes and cultured CTCs of the four patients using the AllPrep DNA/RNA Mini Kit. WES libraries were generated using the Agilent SureSelect Human All Exon V6 kit (Agilent Technologies, CA, USA) according to the manufacturer’s instructions. Double-end (2 × 150 bp) sequencing of WES libraries was performed using the Novaseq 6000 platform. The sequencing depth was 100× for peripheral blood leukocytes (approximately 12 Gb per sample) and 200× for tumor tissue and CTCs samples (approximately 24 Gb per sample). Sequencing data were filtered by adapters and low-quality reads were filtered by FASTQ (v0.12.6). GATK (v3.6) detects SNV. Valid sequencing data were aligned with the human reference genome (GRCh37/hg19) by BWA-MEM (v0.7.12), sorted by SAMtools comparison, and tagged with duplicate reads by Sambamba. Finally, the coverage, depth, etc., of the duplicate reads were calculated using the duplicate tagging results.

We controlled the quality of the primary focal tumor, CTC and CDX models data from four patients using fastp (v0.12.6) to remove low sequencing quality and splice sequences; BWA (v0.7.5a) to match the data to the hg19 reference gene; Mutect2 (GATK3.8) to complete variant detection; Xenome (v1.0.1-r) was used to capture the human margin data from CDX samples before performing the above data analysis. The RNA-seq expression and survival status of kidney renal clear cell carcinoma (KIRC) patients were then downloaded from the Cancer Genome Atlas (TCGA) database (https://tcga-data.nci.nih.gov/tcga/, accessed date on 15 January 2023). Subsequently, the TCGA RCC cohort (TCGA-KIRC) was further used to validate the correlation between the gene expressions and prognosis in RCC patients. We calculated the expression differences between normal and tumor samples using a R software (version 4.2.2) and used unpaired Wilcoxon Rank Sum and Signed Rank Tests for significance of differences analysis with false discovery rate (FDR) < 0.05 and |log2FC| > 1 as the selection criteria.

## 3. Results

### 3.1. Successful Preparation of Synthetic Biological Scaffolds

**Figure 1:**
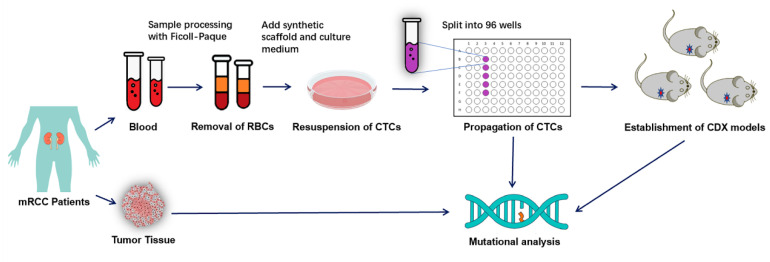
The flowchart provides a comprehensive description of our study, including CTC treatment culture, construction of CDX models and detection and analysis of gene mutations in different samples.

**Figure 2:**
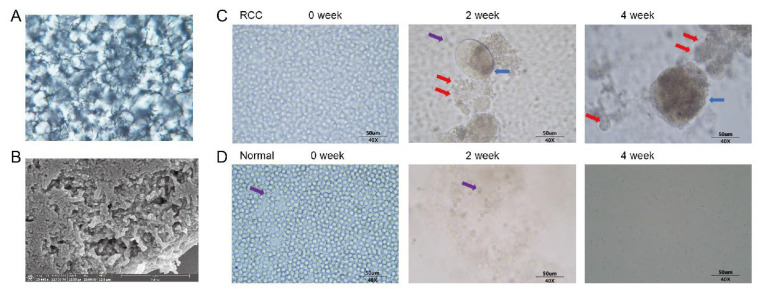
Successful proliferation of CTCs in primary diagnosed mRCC. (**A**) Synthetic scaffold morphology observed by light microscopy; (**B**) Synthetic scaffold morphology observed by electron microscopy; (**C**) Representative phase contrast microscopy images of CTCs from mRCC patients were taken on the first day of culture, day 14 and day 28. The blue arrow indicates circulating tumor endothelial cells (CTECs) which can self-assemble and proliferate into spheres. The red arrow indicates that CTCs attach and proliferate around the endothelial cell spheres. The purple arrow indicates that the leukocytes in the blood then become gradually apoptotic. Scale bars, 50 μm; (**D**) Representative phase contrast microscopy images of healthy subjects were observed in the same way. Under this culture condition, no cells survived in healthy subjects, and residual leukocytes also became gradually apoptotic within 30 days. The purple arrow indicates that the leukocytes in the blood then become gradually apoptotic. Scale bars, 50 μm.

Flowchart Figure 1 provides a comprehensive description of our study, including the CTC treatment culture, the construction of CDX models and the detection and analysis of gene mutations in different samples. Our team successfully constructed synthetic biological scaffolds based on previously applied techniques and enabled copolymer scaffolds to form hydrogel matrices. Hydrogels are formed by cross-linking hydrophilic polymers through covalent bonds or physical attraction. Hydrogels absorb water and swell, making them more porous and permeable for cell migration and the efficient transport of oxygen and nutrient molecules, and they further provide sufficient space for cell proliferation and growth within their matrices. The scaffold images observed by optical microscope and electron microscope are shown in Figure 2A,B.

### 3.2. Proliferation of Peripheral Blood CTCs in mRCC Patients and Healthy Subjects

To culture peripheral blood CTCs from mRCC patients, we performed density gradient centrifugation, CTCs enrichment, addition of biosynthetic scaffolds, and resuspension of CTCs after obtaining blood samples from patients and then cultured the CTCs for up to 30 days. Representative phase contrast microscopy images of CTCs from mRCC patients were taken on the first day of culture, day 14 and day 28. The images showed that distinct circulating tumor endothelial cells (CTECs) are visible on day 14 and day 28, distributed in cell clusters, which can self-assemble and proliferate into spheres. Also visible are distinct CTCs that attach and proliferate around the endothelial cell spheres. Leukocytes in the blood then become gradually apoptotic (Figure 2C). Representative phase contrast microscopy images of healthy subjects were observed in the same way. Under this culture condition, no cells survived in healthy subjects, and residual leukocytes also became gradually apoptotic within 30 days (Figure 2D).

### 3.3. CDX Models Construction and Whole Exome Sequencing

**Figure 3:**
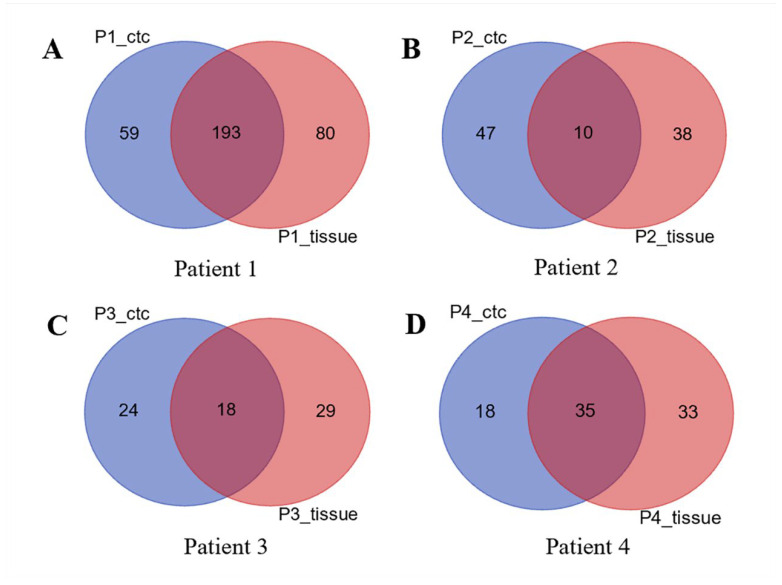
Venn diagrams were used to compare the difference in the number of gene mutations between cultured CTCs and primary tumor tissues. The results showed that both CTCs and tissue type-specific mutated genes were more significantly different; 193 mutated genes shared by Patient1_CTC and Patient1_tissue (**A**); 10 genes shared by Patient2_CTC and Patient2_tissue (**B**); 18 genes shared by Patient3_CTC and Patient3_tissue (**C**); 35 genes shared by Patient4_CTC and Patient4_tissue (**D**).

Cultures of CTCs from four patients and their matched tumor tissues were used for DNA extraction and WES. We then used Venn diagrams to compare the difference in the number of gene mutations between cultured CTCs and primary tumor tissues (Figure 3).

The results showed that both CTCs and tissue type-specific mutated genes were more significantly different; 193 mutated genes shared by Patient1_CTC and Patient1_tissue (Figure 3A); 10 genes shared by Patient2_CTC and Patient2_tissue (Figure 3B); and there were 18 and 35 shared genes for patient 3 (Figure 3C) and patient 4 (Figure 3D), respectively.

**Figure 4:**
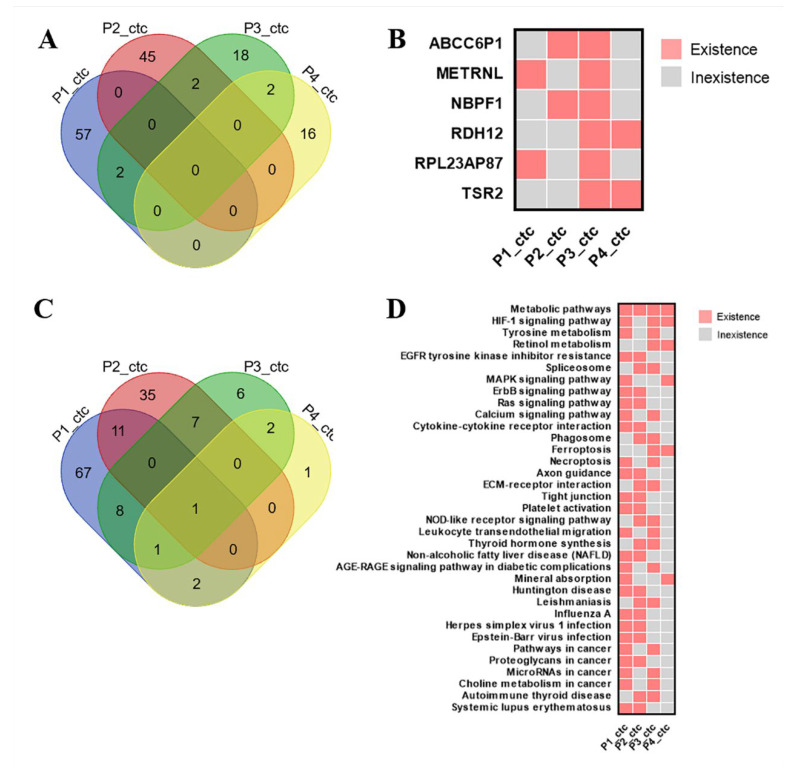
Venn diagrams were used to count the similarities and differences between CTCs type mutation genes in the four samples. From the above CTC differential quantity genes (P1_CTC, P2_CTC, P3_CTC, P4_CTC) we screened out 59, 47, 24 and 18 mutated genes (**A**). Among them, cluster analysis showed ABCC6P1 and NBPF1 shared by P2_CTC and P3_CTC, METRNL and RPL23AP87 shared by P1_CTC and P3_CTC, RDH12 and TSR2 shared by P3_CTC and P4_CTC (**B**). Venn diagrams were used to count the similarities and differences in the KEGG pathway for the specific mutated genes mentioned above. Individual differences in the pathways were also significant. From the KEGG analysis (P1_CTC, P2_CTC, P3_CTC, P4_CTC) we screened out 90, 58, 25 and 7 pathways (**C**). Among them, cluster analysis showed metabolic pathways shared by P1_CTC, P2_CTC, P3_CTC and P4_CTC, HIF-1 signaling pathway shared by P1_CTC, P3_CTC and P4_CTC. MAPK signaling pathway and ErbB signaling pathway were also present in the CTC type data pathway in the present four samples (**D**).

Meanwhile, we used Venn diagrams to count the similarities and differences of CTCs type mutation genes in the four samples (Figure 4A). The results showed that the genes specific to each of the four samples differed significantly, and only individual genes were identical to the other samples. The individual differences in the current four samples were great. From the above CTC differential quantity genes (P1_CTC, P2_CTC, P3_CTC, P4_CTC), we screened out 59, 47, 24 and 18 mutated genes. Among them, ABCC6P1 and NBPF1 were shared by P2_CTC and P3_CTC, METRNL and RPL23AP87 were shared by P1_CTC and P3_CTC, RDH12 and TSR2 were shared by P3_CTC and P4_CTC (Figure 4B).

We used a Venn diagram to count the similarities and differences in the KEGG pathway for the specific mutated genes mentioned above (Figure 4C). Individual differences in the pathways were also significant. From the KEGG analysis (P1_CTC, P2_CTC, P3_CTC, P4_CTC), we screened out 90, 58, 25 and 7 pathways. Among them, metabolic pathways shared by P1_CTC, P2_CTC, P3_CTC and P4_CTC, HIF-1 signaling pathway were shared by P1_CTC, P3_CTC and P4_CTC. The MAPK signaling pathway and ErbB signaling pathway were also present in the CTC type data pathway in the present four samples, but the differences in the samples appearing suggest that there are individual differences in these common pathways as well (Figure 4D).

**Figure 5:**
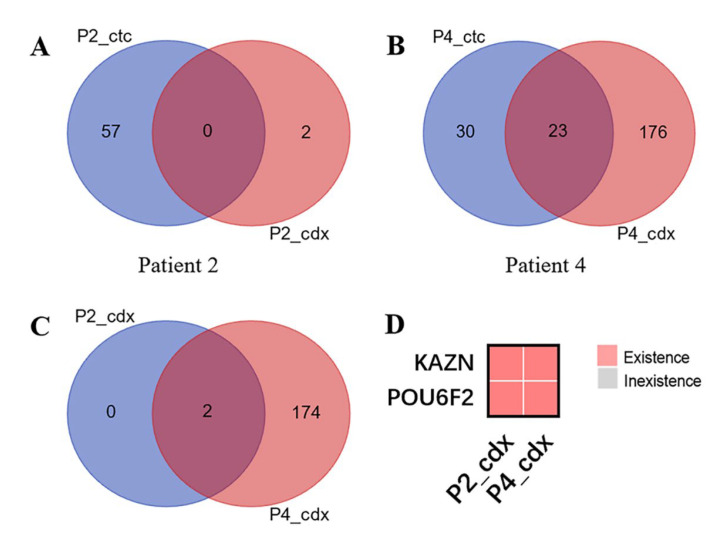
The similarities and differences between CDX type mutated genes in the two samples were counted using Venn diagrams. The results showed that the CDX types of the different samples also reflected some individual differences; only two mutated genes were in the P2_CDX (**A**). Some 23 mutated genes were shared by P4_CTC and P4_CDX (**B**). Two genes were shared by Patient2_CDX and Patient4_CDX (**C**). Cluster analysis showed that these two differentially mutated genes KAZN and POU6F2 also existed in both P2_CDX and P4_CDX (**D**).

Finally, we used the WES to compare the differences in the number of mutated genes between CDX and CTC. The similarities and differences between CDX-type mutated genes in the two samples were counted using Venn diagrams (Figure 5). The results showed that the CDX types of the different samples also reflected some individual differences; only two mutated genes were in the P2_CDX (Figure 5A). Some 23 mutated genes were shared by P4_CTC and P4_CDX (Figure 5B). However, these two differentially mutated genes KAZN and POU6F2 also existed in both P2_CDX and P4_CDX (Figure 5C,D).

### 3.4. Bioinformatics Analysis Results of KAZN and POU6F2

**Figure 6:**
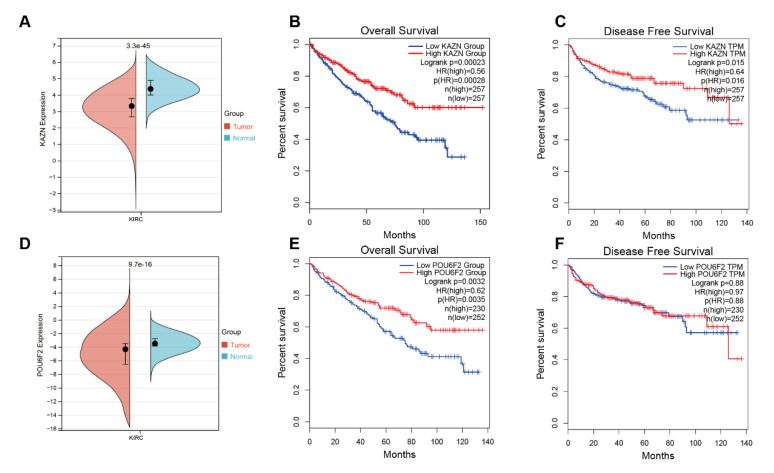
KAZN was highly expressed in normal tissues compared with tumor tissue in the TCGA-KIRC cohort (*p* = 3.3 × 10^−45^) (**A**). Survival analysis showed that high expressions of KAZN had a significantly worse overall survival (OS) rate (*p* = 0.00023) (**B**) and disease-free survival (DFS) rate (*p* = 0.015) (**C**) compared to patients with low expressions of KAZN. POU6F2 was also highly expressed in normal tissue compared with tumor tissue (*p* = 9.7 × 10^−16^) (**D**) and patients with high expressions of POU6F2 had a significantly worse overall survival (OS) rate (*p* = 0.0032) compared to patients with low expressions of POU6F2 (**E**), but this was not statistically significant in DFS (**F**).

We then validated the correlation between the two mutated genes and prognosis in the TCGA-KIRC cohort. The results showed that KAZN was highly expressed in normal tissue compared with tumor tissue (Figure 6A) and patients with high expressions of KAZN had a significantly worse overall survival (OS) rate (*p* = 0.00023) and disease-free survival (DFS) rate (*p* = 0.015) compared to patients with low expressions of KAZN (Figure 6B,C). POU6F2 was also highly expressed in normal tissue compared with tumor tissue (Figure 6D) and patients with high expressions of POU6F2 had a significantly worse overall survival (OS) rate (*p* = 0.0032) compared to patients with low expressions of POU6F2 (Figure 6E), but this was not statistically significant in DFS (Figure 6F).

## 4. Discussion

Blood-borne dissemination is an important way of RCC metastasis, and the entry of tumor cells into peripheral blood is a prerequisite for distant tumor metastasis [19]. In 1867, the Australian medical scientist Ashworth first discovered blood cells similar to tumor cells in the peripheral blood of a deceased tumor patient, thus introducing the concept of CTC [20]. In recent years, studies have shown that CTC is a necessary condition for the invasion and distant metastasis of RCC [9,10]. The small number of CTCs in peripheral blood makes precise isolation difficult, limiting the application of CTCs. In this study, we successfully cultured and amplified CTCs in vitro for the first time in the world from patients with primary diagnosed mRCC, and then inoculated CTCs subcutaneously in nude mice to construct a CDX model. Our research results maybe highlight the satisfactory clinical application value of CTCs in RCC.

Studies have confirmed that a significant proportion of cancer patients have metastatic recurrence within five years after a resection of the primary tumor without detecting metastasis during follow-up, indicating that these patients have minimal residual disease (MRD) [21], which leads to the proposal of “liquid biopsy” of CTCs in the blood for early tumor detection, recurrence monitoring, efficacy testing and to find new therapeutic targets [22]. CTCs are viable tumor cells that can undergo single cell sequencing and whole transcriptome analysis, thus providing patients with a more real-time and dynamic detection effect, which shows immeasurable application in a variety of tumor research fields [23,24]. A study showed that a transcriptional profile detectable in CTCs obtained from liquid biopsies can serve as an independent prognostic marker beyond AR-V7 in patients with metastatic prostate cancer and can be used to identify the emergence of multiple androgen receptor signaling inhibitors (ARSIs) resistance mechanisms [25]. CTCs are also an independent predictor of shorter survival in patients undergoing a resection for pancreatic and periampullary adenocarcinoma [26]. Similarly, CTC has shown very promising value in tumors such as gastric cancer, colorectal carcinoma, and breast cancer [27,28,29].

Due to the low expression of CTC-related immune markers and the high heterogeneity of RCC, the current field of CTC research in RCC still needs further exploration. With the rise and development of high-throughput next generation sequencing (NGS) in recent years, WES has been increasingly used in the diagnosis of RCC [30]. However, practical WES data on the integration of CTCs for RCC with clinical applications are still extremely limited. In the present study, we performed WES and explored some of the mutated genes based on CTCs culture.

NBPF1 plays a bidirectional role in cancer [31]. On the one hand, the tumor suppressive function of NBPF1 has been observed in prostate cancer, neuroblastoma, cutaneous squamous cell carcinoma and cervical cancer [32,33,34]. On the other hand, it has been shown that NBPF1 regulates colony formation and the invasion of hepatocellular carcinoma cells, thus acting as an oncogene [35]. A pan-cancer study obtained the information that NBPF1 is differentially expressed in a variety of cancers including RCC. Elevated levels of NBPF1 expression in KIRC patients corresponded to better OS and DFS. Reduced NBPF1 mRNA expression was also associated with shorter OS in KIRC by univariate Cox regression analysis [31].

KAZN encodes a protein that plays a role in desmosome assembly, cell adhesion, cytoskeletal organization, and epidermal differentiation [36]. This protein co-localizes with desmoplakin and the cytolinker protein periplakin. In general, this protein localizes to the nucleus, desmosomes, cell membrane, and cortical actin-based structures. Some isoforms of this protein also associate with microtubules [36,37]. Alternative splicing results in multiple transcript variants encoding distinct isoforms. Studies have also shown that KAZN was up-regulated in ovarian epithelial tumors and the expression of KAZN was correlated with the patients’ survival time [38]. 

POU6F2 encodes a member of the POU protein family characterized by the presence of a bipartite DNA-binding domain, consisting of a POU-specific domain and a homeodomain, separated by a variable polylinker. The DNA-binding domain may bind to DNA as monomers or as homo- and/or heterodimers, in a sequence-specific manner. The POU family members are transcriptional regulators, many of which are known to control cell type-specific differentiation pathways [39]. A study revealed that POU6F2 may play a crucial role in the development of prolactinomas and may be a promising target for developing new therapies against prolactinomas [40]. In this study, we identified the two differentially mutated genes KAZN and POU6F2 that also existed in both P2_CDX and P4_CDX and confirmed that high expressions of KAZN and POU6F2 had a significantly worse OS rate, which was maybe closely related to the prognosis of RCC. 

There are still some limitations in our study. Firstly, the number of patients in this study cohort is insufficient, which needs to be further verified in more data. Secondly, our study only focused on the potential driver gene mutations which may promote RCC metastasis and implantation based on CTCs culture. Further studies are needed to clarify the mechanism of those genes involved in malignancy progression and metastasis in RCC. 

## Figures and Tables

**Table 1:** Clinicopathological features of 15 ccRCC patients with CTCs culture.

Patient ID	Age	Gender	TNM Staging	IMDC Score *	Grade	Culture Outcome
Patient-01	57	male	T4N0M1	0	2	fail
Patient-02	61	male	T3N0M1	1	2	fail
Patient-03	45	male	T2N0M1	1	1	fail
Patient-04	58	male	T3N0M1	1	2	succeed
Patient-05	50	male	T3N0M1	1	3	succeed
Patient-06	67	male	T1N1M1	1	2	fail
Patient-07	48	male	T4N1M1	3	2	succeed
Patient-08	73	male	T2N0M1	1	4	succeed
Patient-09	63	male	TxN1M1	1	3	succeed
Patient-10	70	male	T3N1M1	1	2	succeed
Patient-11	55	male	TxN0M1	1	3	succeed
Patient-12	54	male	T4N1M1	2	2	fail
Patient-13	73	male	T3N0M1	1	4	succeed
Patient-14	48	male	T1aN1M1	1	4	succeed
Patient-15	50	male	T3N0M1	0	2	succeed

* IMDC score: International Metastatic Renal Cell Carcinoma Database Consortium score.

## Data Availability

The data used and analyzed during the current study are available from the corresponding author upon reasonable request.
